# Effects of graded levels of dietary protein supplementation on milk yield, body weight gain, blood biochemical parameters, and gut microbiota in lactating ewes

**DOI:** 10.3389/fvets.2023.1223450

**Published:** 2023-08-03

**Authors:** Xiaoqi Zhao, Sikandar Ali, Mohammad Farooque Hassan, Muhammad Amjad Bashir, Xiaojun Ni, Chunrong Lv, Hongyuan Yang, Baiji Danzeng, Guobo Quan

**Affiliations:** ^1^The Small Ruminant Department, Yunnan Animal Science and Veterinary Institute, Kunming, Yunnan, China; ^2^Zhejiang Vegamax Biotechnology Co., Ltd., Huzhou, China; ^3^Department of Animal Nutrition, Shaheed Benazir Bhutto University of Veterinary and Animal Sciences, Sakrand, Sindh, Pakistan; ^4^Department of Plant Protection, Faculty of Agricultural Sciences, Ghazi University, Dera Ghazi Khan, Punjab, Pakistan

**Keywords:** lactating ewes, weight gain, fecal microbiomes, dietary protein, blood biochemical parameters, milk yield

## Abstract

Diet-associated characteristics such as dietary protein levels can modulate the composition and diversity of the gut microbiota, leading to effects on the productive performance and overall health of animals. The objective of this study was to see how changes in dietary protein levels affect milk yield, body weight gain, blood biochemical parameters, and gut microbiota in lactating ewes. In a completely randomized design, eighteen ewes were randomly assigned to three groups (*n* = 6 ewes/group), and each group was assigned to one of three dietary treatments with different protein contents. The ewes' groups were fed on 8.38% (S-I), 10.42% (S-m), and 13.93% (S-h) dietary protein levels on a dry basis. The body weight gain and milk yield were greater (*p* < 0.05) in ewes fed the S-h dietary treatment than in those fed the S-m and S-1 diets, respectively. However, milk protein contents were similar (*p* > 0.05) across the treatments. The blood glucose, total protein, cholesterol, triglycerides, high-density lipoprotein, low-density lipoprotein, lactate, creatinine, and C-reactive protein contents of lactating ewes were not influenced (*p* > 0.05) by different dietary protein levels. The alanine transaminase, aminotransferase, and lactate dehydrogenase activities were also not changed (*p* > 0.05) across the groups. However, blood urea nitrogen and albumin contents of lactating ewes were changed (*p* < 0.05) with increasing levels of dietary protein, and these metabolite concentrations were higher (*p* < 0.05) for S-h than the rest of the treatments. In the different treatment groups, Firmicutes and Bacteroidetes were found to be the most dominant phyla. However, the abundance of *Lachnospiraceae* species decreased as dietary protein levels increased. Within the *Bacteroidetes phylum, Rikenellaceae* were more abundant, followed by *Prevotellaceae*, in ewes fed the S-m diet compared to those fed the other diets. Based on the results, feeding at an optimal protein level improved milk yield and body weight gain through modifying the digestive tract's beneficial bacterial communities. The results of blood metabolites suggested that feeding higher-protein diets has no negative impact on health.

## 1. Introduction

Milk has been an important food source for human societies for thousands of years, and the domestication of animals played a significant role in the development of dairy products (1, 2). The first domesticated animals were sheep and goats, which were easy to manage and provided milk, meat, and wool (3–5). The history of sheep farming in China dates back thousands of years, with the Yunnan Semi-fine wool sheep being one of the important cultural breeds (5). This breed is known for its excellent wool quality, high adaptability, and strong robustness, making it suitable for wool and meat production (6). Over the years, efforts have been made to improve the wool quality of Yunnan Semi-fine wool sheep through crossbreeding with other breeds such as Rambouillet hybrid sheep, Caucasian sheep, Xinjiang fine-wool sheep, New Zealand Romney sheep, and Lincoln sheep (7). These efforts have resulted in the development of an ideal cross combination that has helped to increase the population of Yunnan Semi-fine wool sheep (6, 7). The high adaptability of Yunnan Semi-fine wool sheep to cold climates, high altitudes, and hypoxic conditions has made them a preferred choice for sheep farming in alpine regions (8) and likewise, adaptability was also observed in other domestic animals (9). As a result, the population of Yunnan Semi-fine wool sheep has been continuously expanding, and they have become an important source of wool and meat products in China.

Protein is an essential nutrient for the growth, development, and maintenance of tissues and cells in the body. As a nitrogen donor, it plays a crucial role in the synthesis of hormones, enzymes, and other essential molecules (10). Inadequate protein intake can lead to malnutrition, weakness, and disease in lactating ewes. The deficiency can also affect the growth and development of their lambs, leading to stunted growth and reduced immunity. On the other hand, excessive protein intake can overload the metabolism of the ewe and result in the deamination of excess amino acids. The by-products of deamination, such as ammonia and urea, can be toxic and cause health problems. Moreover, the excess nitrogen excreted in urine and feces can lead to environmental pollution and damage ecosystems (10–12). Therefore, it is essential to provide adequate but not excessive amounts of protein in the diet of lactating ewes to maintain their health and prevent environmental pollution. Sheep performance traits can be improved through better breeding plans, proper nutrition, modern management and good health (13). Sheep usually eat outside in the summer and in the barn in the winter, depending on the weather. Sheep are being raised indoors, so their eating habits are becoming more consistent. So, the effects of feeding on sheep production became an important area of research (13, 14). Proteins are essential nutrients for ruminants, and their metabolic activities and production depend on the quality and quantity of proteins in their diet (14, 15). Sheep milk is considered to have higher nutritional value than goat and cow milk, with higher levels of proteins, lipids, minerals, and vitamins that are essential to human health (16, 17).

The rumen microbiota plays a critical role in degrading volatile fatty acids, vitamins, and microbial proteins, providing almost 3rd quarter of the energy requirement in the sheep body (18). Early feeding and the quality and nutritional composition of feed can impact the rumen microbiome, which can ultimately affect the animal's performance (19). The study aims to investigate the effect of different crude protein dietary levels on milk yield, body weight gain, blood biochemical parameters, and the abundance of fecal microbiome populations in lactating Yunnan semi-fine wool sheep.

## 2. Materials and methods

### 2.1. Ethics statement

The current study on Ewes was approved by the ethical committee of Yunnan Animal Science and Veterinary Institute (201911004). Proper protocols and guidelines were followed during the field trials as per (Order-No.2 of the State Science and Technology Commission of the People's Republic of China, 1988) and (the Standing Committee of Yunnan Provincial People's Congress 2007.10).

### 2.2. Preparation of Ewes for the experiment, measurement of body weight, and collection of fecal samples

The experiment site (26° 22N; 103° 40E) is at Yunnan Animal Science and Veterinary Institute, Kunming City, China (26° 22N; 103° 40E). For this purpose, a total of 18 (2-year-old) ewes with an average body weight of 38.52 ± 1.57 kg were divided in to 3 groups and each group was consisted of 6 ewes for the experimental trial. The feed was formulated according to the previous study with slight modification 20 and various protein levels were fed to experimental animals as shown in Table 1. After 135 days of pregnancy, the ewes were introduced to the formulated diet twice daily at precisely 8:30 a.m. and 16:00 p.m. The ewes were divided into three groups (S-I, S-m, and S-h) and fed diets with different protein levels such as 8.58, 10.34, and 13.93%, respectively. The formulated diet was given twice a day, and corn silage was given to ewes at other times of the day. The ewes were housed individually and had *ad libitum* access to water. The fecal samples were collected from the terminal rectum after 90 days of parturition, and ~10 g of fresh fecal samples were stored in a 10 ml sterile freezing tube and immediately transported to a −80°C freezer for storage. The body weight of the ewes was recorded at the beginning of the experiment, after 12 h of parturition, and on the 90th day of the study. The ewes are milked twice a day during feeding time.

**Table 1:** The feed formulation, nutrient contents, and different protein levels (air-dry basis).

**Items**	**S_l**	**S_m**	**S_h**
Corn	28.2	26	19.05
Soybean meal	5.40	8.65	18.60
Corn starch	8.65	7.60	4.70
Calcium carbonate	0.55	0.60	0.65
Calcium hydrogen phosphate	0.55	0.50	0.35
Salt	0.30	0.30	0.30
Baking soda	0.35	0.35	0.35
Premix	1.00	1.00	1.00
Corn silage	40.00	35.00	34.00
Bean powder	2.00	10.00	11.00
Wheat straw	13.00	10.00	10.00
Total	100	100	100
Fine to coarse ratio	45:55	45:55	45:55
**Nutrient contents**			
Metabolizable energy	9.45	9.47	9.47
Protein	8.58	10.34	13.93
Neutral detergent fiber (NDF)	32.17	32.01	32.52
Acid detergent fiber (ADF)	17.24	17.71	18.44
Calcium	0.69	0.71	0.71
Phosphorus	0.38	0.39	0.39

### 2.3. Blood biochemical parameters

Approximately 5 ml of blood was collected from their jugular vein using vacutainer tubes containing ethylenediaminetetraacetic acid (EDTA), before feeding on the morning of the 90th day. The blood samples were then centrifuged at 4°C for 15 min at 3,000 × g, and the resulting plasma was stored at −20°C until further analysis. The plasma samples were analyzed for various parameters using commercial kits manufactured by Roche Diagnostics Products Co., Ltd., Shanghai, China. The blood parameter studied includes; aspartate aminotransferase (AST), glucose (GLU), total protein (TP), alanine aminotransferase (ALT), blood cholesterol (CHOL), triglyceride (TG), lactate dehydrogenase (LDH), high-density lipoprotein-cholesterol (HDL), low-density lipoprotein-cholesterol (LDL-C), lactic acid (LACT), creatinine (CREA), c-reactive protein (CRP), UREA, blood urea nitrogen (BUN), and albumin (ALB).

### 2.4. DNA extraction

According to the manufacturer's instructions, DNA was extracted from the fecal samples using the E.Z.N.A.^®^ Stool DNA Kit. After the DNA extraction process, the DNA was eluted using 50 μL of the Elution buffer. Elution is the process of removing the DNA from the extraction matrix and placing it in a buffer solution, which can then be used for downstream applications such as PCR or sequencing. The Elution buffer used in this protocol likely contains a low salt concentration to minimize interference with downstream applications. Following elution, the DNA samples were stored at −80°C for later use.

### 2.5. PCR amplification and 16S rDNA sequencing

The amplification and sequence of the V3-V4 region of the prokaryotic small-subunit (16S) rRNA gene was performed by using specific primer 341F (5′-CCTACGGGNGGCWGCAG-3′) and 805R (5′-GACTACHVGGGTATCTAATCC-3′). This gene is commonly used as a marker for identifying and classifying bacteria and archaea. The primers were modified with barcodes and universal primers to enable multiplexing and sequencing of multiple samples. The PCR reaction mixture contained a 25 μL reaction mix containing 25 ng template DNA, 2.5 μL of each primer, 12.5 L of PCR premix, and PCR grade water used to adjust the total volume for PCR amplification. The PCR reaction mixture contained template DNA, primers, PCR premix, and water, and the reaction was run for 32 cycles with specific temperature conditions for denaturation, annealing, and extension. The final PCR products were evaluated using 2% agarose gel electrophoresis, and then purified and quantified using AMPure XT beads and Qubit, respectively. The size and quantity of the amplicon library were analyzed using the Agilent 2100 Bioanalyzer, and the library was quantified using the Illumina library quantification kit. Finally, the libraries were sequenced using the NovaSeq PE250 platform.

### 2.6. Bioinformatic analysis

The acquired raw sequencing reads were processed to obtain valid reads for further analysis. In the first step, cutadapt v1.9 is used to remove these sequencing adapters from the raw sequencing reads 21. Sequencing adapters are short pieces of DNA that are added to the ends of DNA fragments to allow them to be sequenced by the sequencing machine. This is important because adapters can cause problems during downstream analysis, such as errors in read alignment and variant calling. After adapter removal, the low-quality reads are trimmed using fqtrim v0.94. This step removes low-quality bases from the ends of reads, which can improve the accuracy of downstream analysis. The trimming is done using a sliding-window algorithm, which calculates the average quality score over a fixed window size and trims bases with scores below a certain threshold. The remaining reads are then aligned to the host genome using bowtie2 22. This step removes any reads that match to the host genome, which can be useful when analyzing microbial samples that are expected to have lower host contamination.

### 2.7. Statistical analysis

The statistical analysis was performed by using the SPSS 19.0 software. One-way ANOVA was used to analyze data related to daily gain, Chao1, observed species, and Shannon index. The Tukey test (HSD) was used to compare means, and the results were expressed as means ± SEM. The cutoff for statistical significance was set at *P* < 0.05 or *P* < 0.01, indicating that any observed differences with a *P*-value less than these thresholds were considered statistically significant.

## 3. Results

### 3.1. Effect of different dietary protein levels on weight gain, milk yield, and protein content in sheep

**Figure 1:**
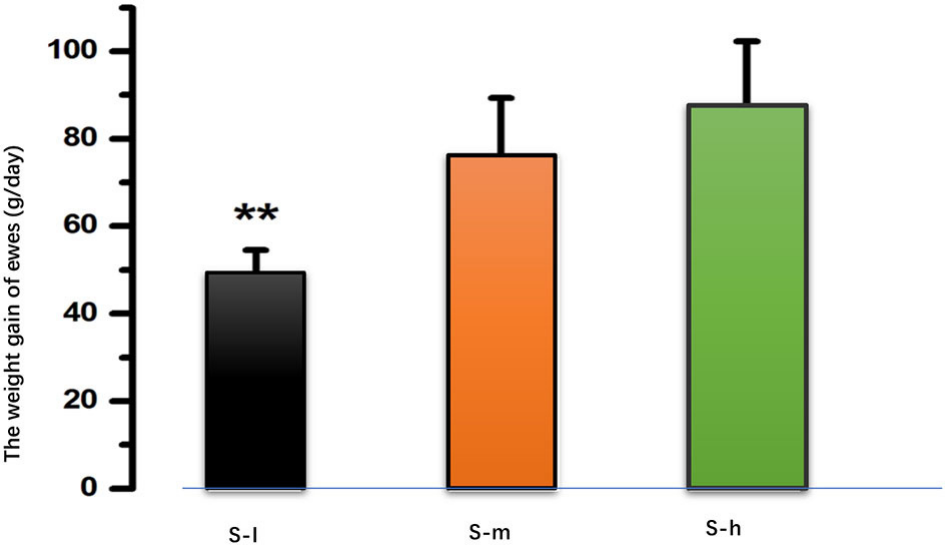
After 90 days of feeding, the mean daily gain in the S-I group (49.065.47 g/day) was significantly lower than in the S-m group (76.2413.10 g/day, *P* < *0.01*) and the S-h group (87.5514.78 g/day, *P* < *0.01*). However, there is no difference between the S-m group and the S-h group (*P* > *0.01*). ^**^means highly significant (*p* < 0.01).

The daily weight gain of lactating ewes was measured and compared among the three groups. The results showed that there was a significant difference (*P* < *0.01*) in daily weight gain between the S-I group and both the S-m and S-h groups as shown in Figure 1. However, there was no significant difference (*P* > *0.01*) in weight gain between the S-m and S-h groups. This suggests that a higher level of dietary protein may be beneficial for weight gain in lactating ewes. The daily milk yield was also measured and compared among the three groups. The results showed that the milk yield of the S-I group was significantly (*P* < *0.01*) lower than that of the S-m and S-h groups (Table 2). However, there was no significant difference (*P* > *0.01*) in milk yield between the S-m and S-h groups. This suggests that a higher level of dietary protein may also be beneficial for milk yield in lactating ewes. The milk protein content was also measured, but no significant differences (*P* > *0.05*) were found between the groups. Overall, these results suggest that a higher level of dietary protein may be beneficial for weight gain and milk yield in lactating ewes, but may not affect milk protein content.

**Table 2:** Effect of dietary crude protein level on milk yield and milk protein content of Yunnan semi fine wool sheep in late lactation.

**Items**	**S-l**	**S-m**	**S-h**
Milk yield (kg/d)	0.54 ± 0.09^b^	0.76 ± 0.16^a^	0.83 ± 0.20^a^
Milk protein content (%)	5.54 ± 0.50^b^	5.66 ± 0.50^b^	5.96 ± 0.84^ab^

a, b represents differentiation in results.

### 3.2. Impact of different dietary protein levels on the blood biochemical parameters

Based on the results presented in Table 3 and the mentioned studies, it can be concluded that different levels of dietary crude protein had no significant (*P* > *0.05*) effects on the majority of the blood biochemical indexes in sheep, including aspartate aminotransferase (AST), glucose (GLU), total protein (TP), alanine transaminase (ALT), cholesterol (CHOL), triglycerides (TG), lactate dehydrogenase (LDH), high-density lipoprotein (HDL), low-density lipoprotein (LDL), lactate (LACT), Creatinine (CREA), and C-reactive protein level (CRPL). However, the concentration of albumin (ALB) and blood urea nitrogen (BUN) increased linearly as dietary protein intake increased within the groups. The increase in albumin concentration may be related to the modulation of albumin synthesis and catabolism by dietary protein intake, while the increase in BUN concentration may be due to the low energy of supplementary feed or the negative balance of energy and nitrogen in the rumen. Moreover, the concentration of urea in the serum of lactating ewes also increased linearly with the increase of protein level, which may be due to the contribution of ammonia released from the ruminal degradation of dietary or endogenous urea to the maintenance of physiological ruminal pH. However, the highest level of urea in the study was not considered a health hazard for ewes.

**Table 3:** Effect of different dietary crude protein levels on blood biochemical parameters of lactating Yunnan semi fine wool sheep.

**Items**	**S-l**	**S-m**	**S-h**
AST, U/L	110.22 ± 10.12	128.6 ± 11.21	132.9 ± 14.51
GLU, mmol/L	3.17 ± 0.38	2.71 ± 0.28	2.97 ± 0.27
TP, g/L	58.14 ± 3.01	61.04 ± 2.93	66.4 ± 8.66
ALT, U/L	16.29 ± 5.91	17.93 ± 5.99	15.58 ± 4.16
CHOL mmol/L	2.12 ± 0.46	1.98 ± 0.20	1.84 ± 0.35
TG, mmol/L	0.20 ± 0.007	0.25 ± 0.009	0.28 ± 0.011
LDH, U/L	511.55 ± 11.71	499.33 ± 7.55	530.11 ± 9.63
HDL, mmol/L	1.54 ± 0.13	1.42 ± 0.09	1.13 ± 0.15
LDL-C, mmol/L	0.57 ± 0.030	0.48 ± 0.048	0.53 ± 0.044
LACT, mmol/L	4.56 ± 0.44	3.97 ± 0.16	4.33 ± 0.29
CREA, μmol/L	39.94 ± 1.08	49.00 ± 1.24	42.78 ± 1.44
CRPL, mmol/L	4.14 ± 0.06	4.11 ± 0.07	4.23 ± 0.09
UREA, mg/dl	8.53 ± 1.51^c^	12.72 ± 2.12^b^	22.27 ± 2.87^a^
BUN mmol/L	2.33 ± 0.29^b^	2.68 ± 0.54^b^	4.98 ± 0.87^a^
ALB, g/L	29.81 ± 2.46^b^	32.05 ± 1.5^ab^	33.82 ± 0.83^a^

a, b, c represents differentiation in results.

### 3.3. Sample clustering analysis

**Figure 2:**
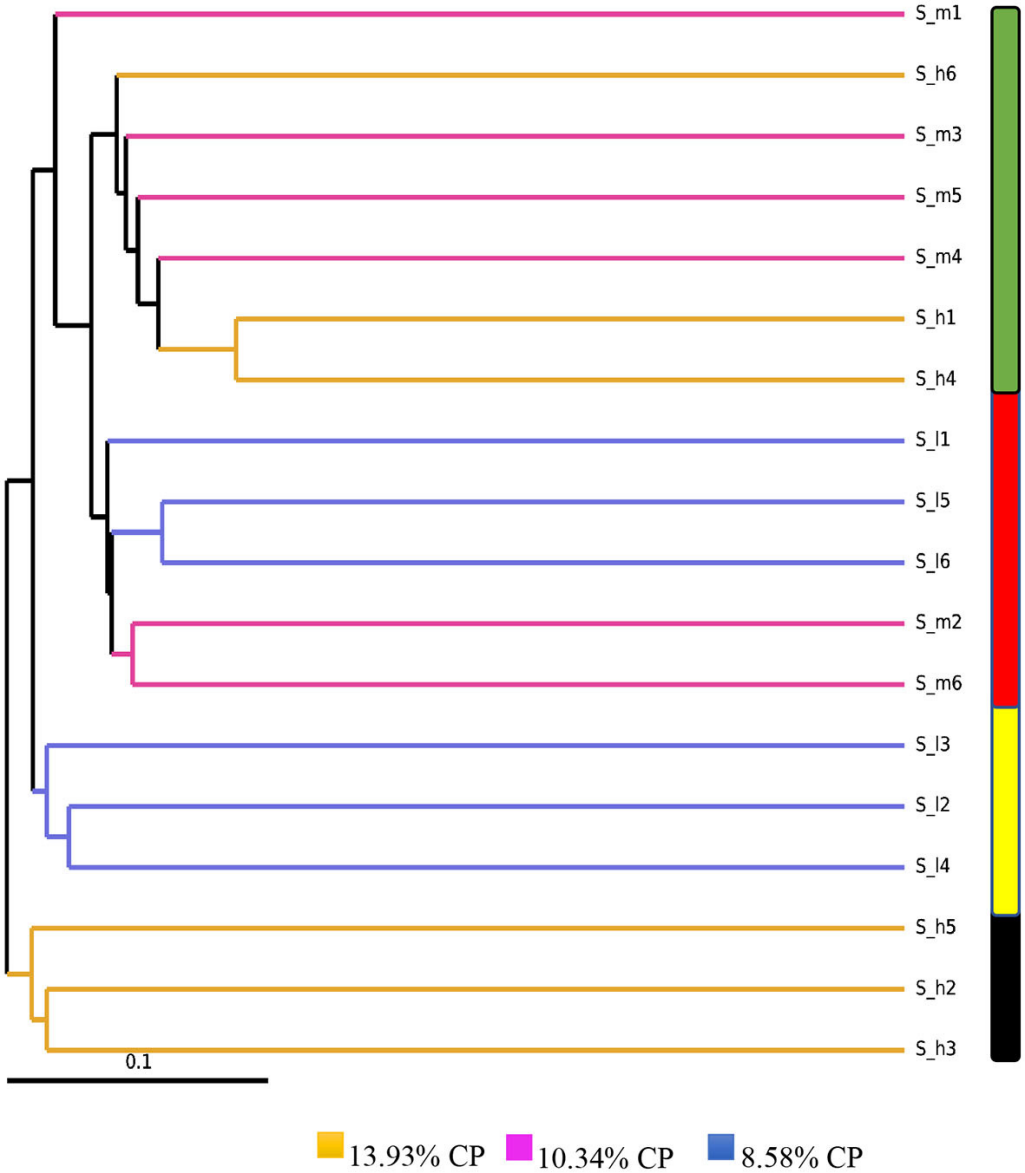
UPGMA clustering based on 18 stool samples from three different levels of dietary crude protein. Each dietary protein group consists of 6 different lactating ewes. Different colors of the branches represent different levels of dietary crude protein.

Based on the analysis of the 16S rDNA sequencing data, we used the unweighted UniFrac index to measure the difference coefficient between the samples. This index takes into account the presence or absence of different microbial taxa in each sample and calculates the similarity between them based on the evolutionary distance of the taxa. As shown in Figure 2, the different clusters are represented by bars of different colors. Samples that are clustered together have a higher similarity and are more likely to have similar microbial communities. The black color bar represents samples S-h2, S-h3, and S-h5, which belong to the same group and have been given the same level of dietary protein. Similarly, the yellow color bar represents samples S-H2, S-I3, and S-I4, which also belong to the same group. However, the clustering of samples may not always be consistent with the group information, particularly when the number of samples is large. In the case of the red and green color bars, the clusters contain a high number of samples from different groups, indicating that the microbial communities in these samples may not be well-defined by the group information. Finally, the sample S-m1 showed the highest dissimilarity with the black color bar cluster, indicating that it has a significantly different microbial community compared to the other samples in that cluster.

### 3.4. Data acquisition

**Figure 3:**
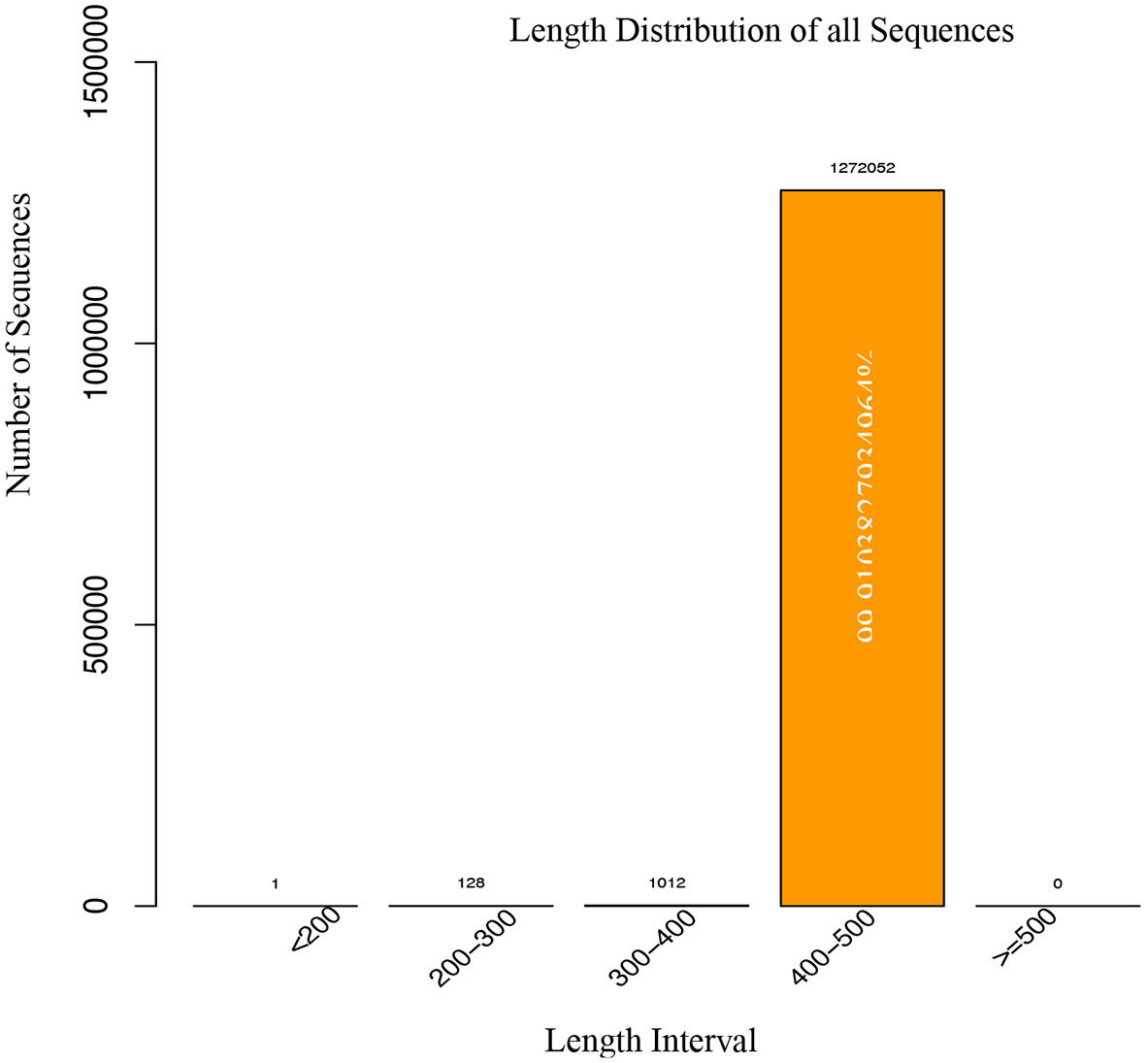
Length distribution of sequences were generated from metagenomics.

The data acquisition process for a study involving 18 fecal samples. A total of 1,514,256 raw tags were obtained, representing 757.12 million raw bases. After filtering for quality and removing chimeras, 1,273,193 valid tags were obtained, representing 521.62 million valid bases. The percentage of valid samples was >80% for all but one sample, which had a percentage of 78.93%. After filtering the chimera, the data were counted and results are depicted in Table 4. We observed that the vast majority (99.99%) of the valid tags were between 400 and 500 nt in length. Sequences with lengths lower than 400 nt were removed, and there were no sequences with lengths higher than 500 nt in the data as shown in Figure 3. The percentage of valid tags with lengths between 300 and 400 nt was 0.07948519980867%, and the percentage with lengths between 200 and 300 nt was 0.0100534640074207%. The length distribution of individual samples is presented in Supplementary Figure 1.

**Table 4:** Valid data statistics of 18 fecal samples originated from lactating Yunnan semi fine wool sheep.

**Sample**	**Raw tags**	**Raw bases**	**Valid tags**	**Valid bases**	**Valid%**	**Q20%**	**Q30%**	**GC%**
S_m1	83,524	41.76M	68,996	28.12M	82.61	98.22	94.27	53.27
S_m2	85,299	42.65M	70,780	29.04M	82.98	98.25	94.16	52.45
S_m3	80,938	40.47M	67,944	27.91M	83.95	98.43	94.78	52.47
S_m4	81,358	40.68M	66,911	27.36M	82.24	98.18	94.11	52.64
S_m5	84,396	42.20M	70,596	28.98M	83.65	96.42	89.53	52.74
S_m6	87,420	43.71M	72,158	29.66M	82.54	98.45	94.93	52.68
S_h1	84,771	42.39M	76,175	31.22M	89.86	98.32	94.61	52.53
S_h2	81,565	40.78M	67,214	27.51M	82.41	97.10	91.34	52.77
S_h3	82,381	41.19M	69,795	28.55M	84.72	98.32	94.45	52.57
S_h4	87,857	43.93M	80,052	32.79M	91.12	98.41	94.83	52.51
S_h5	87,388	43.69M	73,351	30.07M	83.94	97.96	93.50	52.41
S_h6	86,145	43.07M	73,055	29.84M	84.80	98.04	93.91	52.99
S_l1	87,422	43.71M	72,312	29.70M	82.72	97.23	91.56	53.10
S_l2	81,460	40.73M	68,106	27.73M	83.61	98.33	94.64	53.15
S_l3	85,121	42.56M	71,604	29.39M	84.12	97.71	92.79	52.22
S_l4	83,249	41.62M	65,709	26.79M	78.93	96.63	90.09	53.03
S_l5	80,473	40.24M	66,160	27.21M	82.21	98.21	94.12	53.00
S_l6	83,489	41.74M	72,275	29.75M	86.57	98.07	93.67	52.69
Total	1,514,256	757.12M	1,273,193	521.62M				

### 3.5. Abundance and investigation of microbial community

**Figure 4:**
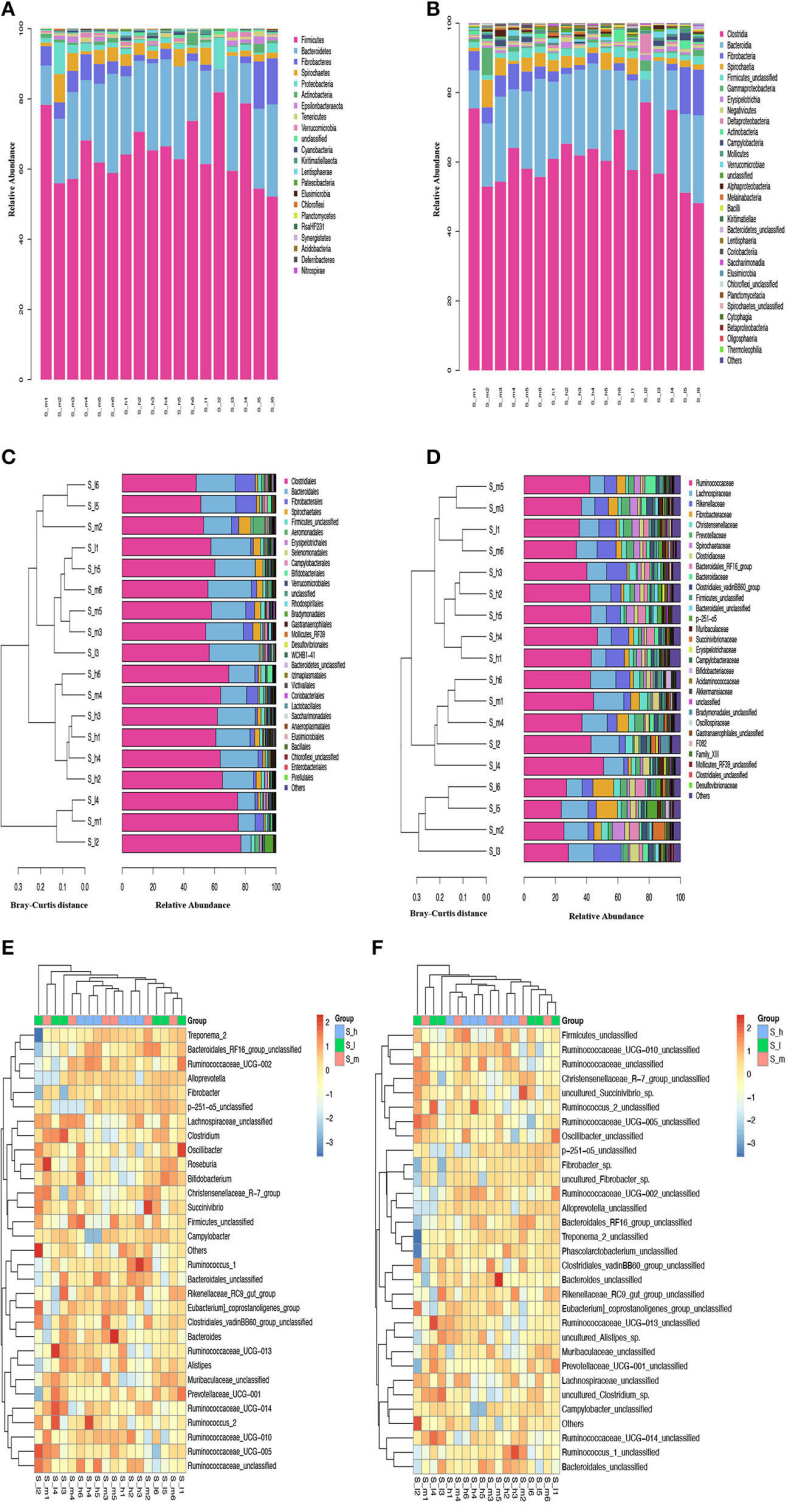
Relative Abundance % of microbiomes **(A)** Phylum, **(B)** Class, **(C)** Order, **(D)** Family, **(E)** Genus, and **(F)** Species in the feces of early lactating Ewes fed graded level of crude protein.

To assess the fecal microbiota in semi-fine wool sheep of Yunnan using metagenome sequencing. The analysis was performed on the V3-V4 region of the 16S rDNA, and the results showed that the microbiota was mainly composed of five phyla with (>2% average relative abundance), namely *Firmicutes* (65.10%), *Bacteroidetes* (21.33%), *Fibrobacteres* (4.15%), *Spirochaetes* (2.86%), and *Proteobacteria* (2.29%) which represents 95.62% of the total composition (Supplementary Table 1 phyla). The results were presented in Figures 4A, B that showed the Phylum and Class of microbes in stacked bar charts, respectively. The microbe with the greatest frequency and frequency across all samples is shown by the color block at the bottom of the column. Other colors reflect strains and classes of bacteria that were recovered from fecal samples taken from sheep. The stacked bar has many columns, each of which represents a separate group.

Among these, *Firmicutes* was the most represented phylum in S-I2 (81.89%) and lowest in S-I6 (52.15%), followed by *Bacteroidetes* that showed that highest (32.75%) and lowest (6.7%) relative abundance in S-I3 and S-I2 groups, respectively. Moreover, the top 30 class taxa, and unclassified class was observed in all groups with highest relative abundance % of 1.03% in S-H2 and lowest in 0.17% in S-H4. The Figures 4C, D represents the order and family of microbes in cluster diagram. The Bray-Curtis distance is used in the sample clustering cluster diagram to describe the degree of similarity between fecal samples. On the left side of the cluster plots is the Bray-Curtis distance of the cluster tree. On the right are the plots of the relative abundance distribution of the species of each sample at the gate level. The larger the proportion, the higher the frequency. The S-I2 sample in Figure 4C was observed at the bottom of the Bray-Curtis distance with the highest relative abundance of 77.19% among the top 30 order taxa. The relative abundance of these phyla and other taxa varied in different treatment groups are presented in Supplementary Table 2.

*Ruminococcaceae* and *Lachnospiraceae* families of *Firmicutes* were the most relatively abundant families in the feces of Yunnan semi-fine wool sheep, and their abundance varied with the level of crude protein in the diet. Similarly, different families of *Bacteroidetes* and *Fibrobacteres* phyla also showed variation in relative abundance. The study also identified unique families and genera specific to certain groups of animals. The analysis was presented in the form of stacked bar charts, cluster diagrams, and heatmaps to demonstrate the abundance and clustering of different taxa Figures 4E, F. According to the relative abundance of each sample, the 30 highest taxa were grouped to the abundance similarity between samples. The gradient from blue to red in the heat map reflects the change in frequency from low to high. The closer to blue, the lower the richness, and the higher the richness when it is close to the red color. The relatively predominant genus found in all groups was *Ruminococcaceae*-UCG-005 from *Ruminococcaceae* (*Firmicutes*) accounting for 10.65% in S-h, 11.69% in S-m and 11.57% in S-I group of all assigned genera included (Figure 4E), within the *Lachnospiraceae* family. The detailed information associated with the abundance at genus levels was included in Supplementary Table 3.

### 3.6. Alpha diversity analysis

**Figure 5:**
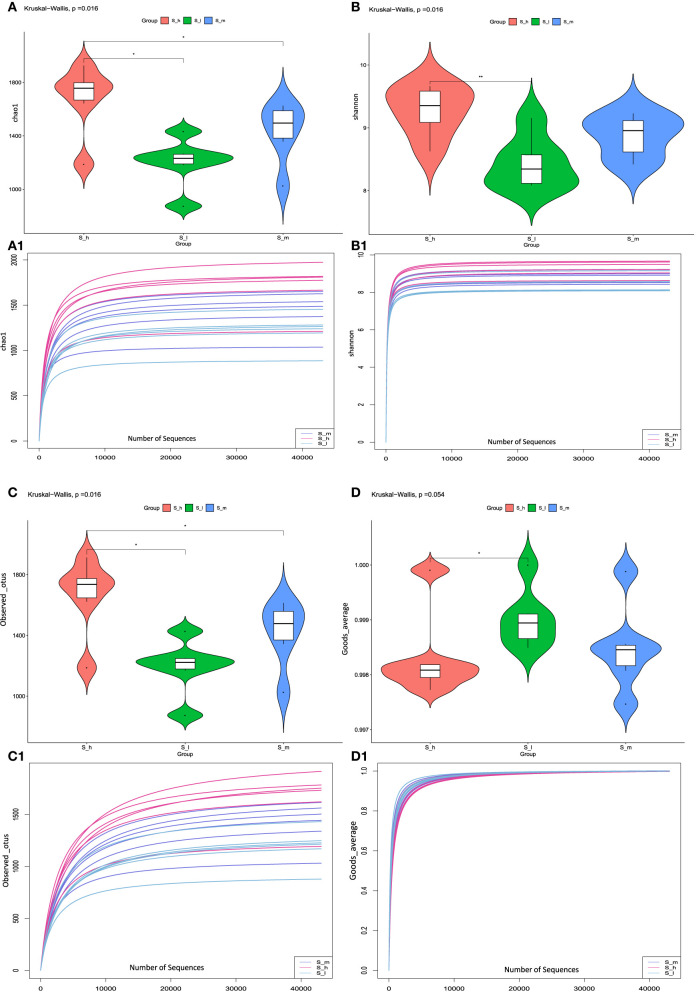
The values related to the chao1, Shannon, observed_otus, and goods_averae index. ^*^means significant (*p* < 0.05) result. ^**^means highly significant (*p* < 0.01) result.

In current study we used alpha diversity analysis to compare microbial diversity in fecal samples treated with three different CP levels. Alpha diversity refers to within-sample diversity or an estimate of species to reflect the richness and evenness of each sample. Several alpha diversity indices were calculated using QIIME, including Chao1 (Figure 5A), Shannon (Figure 5B), Observed_otus (Figure 5C), and Goods_coverage index (Figure 5D). The above index also shows richness and diversity of feces samples treated with three different levels of crude protein in sheep. The results were represented in Violin figures to compare diversity within groups. The rarefaction curve analysis was also performed to analyze diversity indices with respect to the number of randomly selected tags. Rarefaction curves for the indices Chao1 (Figure 5A1), Shannon (Figure 5B1), Observed_otus (Figure 5C2), and Goods_coverage index (Figure 5D1) reveal the microbiological diversity across feces samples. The results of alpha diversity analysis were presented in Supplementary Table 4. The chao1 value and number of observed otus in S-h group were significantly higher than that in S-I and S-m group (*P* < *0.05*), indicating higher microbial richness in S-h group. However, no dissimilarity was found between the S-I group and the S-m group (*P* > *0.05*) in both indexes. The Shannon value in the S-h group was significantly higher in comparison to the S-I group (*P* < *0.01*), suggesting that higher microbial diversity was observed in S-h group. Moreover, the Shannon value in S-m group didn't differ from that of the other two groups (*P* > *0.05*). The value of number of good averages in S-h group was significantly higher (*P* < *0.05*) than that in S-I group. Whereas, non-significant difference was observed between S-m and other two groups. The findings of alpha diversity analysis revealed differences in microbial richness and diversity among the three treatments, with the highest values observed in the S-h group.

## 4. Discussion

Protein is essential for sheep to grow, maintain and repair their tissues, as well as to produce milk during lactation. During pregnancy and lactation, the demand for protein increases significantly, and if the sheep's diet does not provide enough protein, the growth and reproductive performance can be negatively impacted. Feeding a balanced diet that meets the animal's protein requirements can help ensure optimal growth, milk production, and reproductive performance in sheep (13–15). It is interesting to note that adding crude protein to the diet of sheep can have a positive impact on their growth, maintenance, and reproductive performance (14–16). However, the lack of experimental data on the effects of different dietary crude protein levels on body weight, blood biochemistry parameters, abundance of the fecal microbiome and alpha diversity in ewes during the first 90 days after parturition reveals a gap in our understanding of this area. This study found that ewes fed with 10.34% protein level (S-m group) and 13.93% protein level (S-h group) significantly increased the weight gain as compared to the low protein (S-I) level group. The results of this study support the existing body of knowledge and are consistent with previous research findings (20–22). However, there was non-significant difference were observed in respect to weight gain between the regular and higher protein groups. Moreover, the study suggests that an increase in crude protein in the diet can increase milk production as long as the balance of other nutrients is maintained. The groups fed with higher crude protein level in their diet also had increased milk production compared to the low protein group. However, the different crude protein levels did not significantly affect the milk protein content among all the groups, these findings are consistent with the observations made by earlier study (11, 21, 23).

The effect of dietary crude protein (CP) levels on biochemical indexes of sheep is a complex interplay between the specific nutrient composition of the diet and the metabolic processes within the body (24). Diets with higher CP levels are associated with higher concentrations of essential amino acids, which can play a role in regulating metabolic processes (25). Albumin synthesis and catabolism are modulated by dietary protein intake, and albumin provides amino acids to support peripheral tissue anabolism when the dietary protein supply is inadequate to meet requirements (20, 23). The concentration of blood urea nitrogen (BUN) in lactating ewes increased with increasing dietary protein levels, which may be due to low energy of supplementary feed, negative energy and nitrogen balance in the rumen, or inhibition of rumen microbial reproduction (20, 26). However, despite increased BUN and ALB concentration with higher dietary protein levels, the values remained within normal ranges (27, 28). In the current study, urea concentration increased linearly with increasing crude protein intake in lactating sheep groups, but the highest level of urea observed was not pose any health hazard for the ewes (20). Ammonia released from the breakdown of dietary or endogenous urea in the rumen is known to contribute to the maintained the physiological process rumen pH (26, 29). In addition to affecting metabolic processes, high crude protein diets can also impact the microbiome communities in the rumen. A UPGMA clustering phylogenetic tree in the study showed that samples from the high crude protein group clustered separately, indicating differences in microbiome colonies between groups (30). It is important to note that the effect of high crude protein diets on the microbiome communities in the rumen is not fully understood and requires further investigation. Overall, the effect of dietary crude protein levels on biochemical indexes of sheep is dependent on various factors, including the nutrient composition of the diet and metabolic processes within the body. While high crude protein diets can lead to increased BUN and urea concentrations, the levels observed in the current study did not pose a health hazard for the ewes.

The dominance of *Firmicutes* and *Bacteroidetes* among all the phyla in the microbiota of ruminant animals, including sheep, goats, cattle, and pigs, has been reported in several studies (31–37). These two phyla are essential for the microbial ecology of the ruminant gut and have been found to play critical roles in the fermentation of feed components (20, 27, 38). In present study on Yunnan semi-fine wool sheep, the relative abundance of *Firmicutes* was 65.10%, while that of *Bacteroidetes* was 21.33% of the total composition, which is consistent with the findings in sheep feces reported by other studies (18, 39). However, the ratio of *Firmicutes* to *Bacteroidetes* in goats was found to be higher than in sheep (35). In pig fecal samples, *Bacteroidetes* abundance was predominant, ranging from 42.0 to 51.9%, followed by *Firmicutes* (28). In contrast, *Proteobacteria* was dominant in chicken feces, followed by *Firmicutes, Bacteroidetes*, and *Tenericutes* (40). The differences in the abundances of *Firmicutes* and *Bacteroidetes* among these studies may be attributed to various factors such as animal species, growth stages, sampling position and collection time, dietary components, and other environmental factors. In our study, *Ruminococcaceae* within the phylum *Firmicutes* was found to be the most relatively abundant family, representing 42.94% of all the sequences found at the family level. The abundance of *Ruminococcaceae* was higher in the group fed with 13.93% dietary crude protein level compared to the other two groups (36.56 and 34.74%, respectively). In present study we observed that *Rikenellaceae* was the most relatively abundant family in S-m group, followed by *Prevotellaceae*, while *Fibrobacteraceae* was more abundant in S-m and S-I groups compared to S-h group. The above results are consistent with previously reported studies (27, 39). At the genus level, *Ruminococcaceae*-UCG-005 from *Ruminococcaceae* and *Lachnospiraceae* unclassified were the most relatively abundant in all groups, while *Ruminococcaceae*-UCG-010 and *Ruminococcus*-1 showed higher abundance in S-h group compared to S-I group. Conversely, *Fibrobacter* showed the opposite pattern, with higher abundance in S-I group and lower abundance in S-h group. Same kind of pattern was reported in previous studies in respect to the abundance of *Ruminococcus*-1 genus in ruminants (16, 41).

Interestingly, *Ruminococcus* has been suggested to have negative connections with milk production in cows, but a study found an increase in *Ruminococcus* abundance in overweight participants after probiotics intervention compared to normal-weight participants (42, 43). These findings suggest that *Ruminococcus* may have a more complex role in relation to metabolism and weight regulation than previously thought (43, 44). Another study was conducted to assess the effects of probiotics on gut microbiota and their association with childhood obesity (25). The results showed a significant increase in the *Ruminococcus* genus in the obese participants after the probiotic intervention compared to the normal weight participants. Further studies are needed to elucidate the mechanisms underlying these associations and to develop targeted interventions to modulate gut microbiota composition for health benefits.

## 5. Conclusion

In conclusion, the study found that increasing dietary protein levels in early lactating ewes can enhance their daily gain and milk production, as well as increase the levels of ALB and BUN. The lowest level of crude protein was found to be most unfavorable, while the highest level did not significantly outperform the moderate level. The study also revealed that different crude protein levels were associated with gut bacteria and fecal microbiota, with Firmicutes and Bacteroidetes being the dominant phyla in fecal samples regardless of the dietary protein levels. The UPGMA phylogenetic tree showed a distinct cluster of fecal bacteria in the high protein group. Further research is still needed to understand the characteristics of gut bacterial communities at different growth stages. Overall, this study provides valuable insights into the suitable level of crude protein for the health of ewes and their lambs, which can help in the development of effective feeding strategies for early lactating ewes.

## Data availability statement

The data presented in the study are deposited in the NCBI repository, accession numbers SAMN25981821-SAMN25981838.

## Ethics statement

The animal study was reviewed and approved by the Ethical Committee of Yunnan Animal Science and Veterinary Institute (201911004).

## Author contributions

SA and XZ: sampling, investigation, methodology, software, validation, data curation, formal analysis, and writing—original draft. XN and CL: data curation, formal analysis, and investigation. HY and BD: animal management. SA, MH, and MB: revision and writing—review and editing. GQ: conceptualization, funding acquisition, project administration, resources, investigation, supervision, visualization, and writing—review and editing. All authors contributed to the article and approved the submitted version.
