# Supplementary material for: Effects of graded levels of dietary protein supplementation on milk yield, body weight gain, blood biochemical parameters, and gut microbiota in lactating ewes
Source: Front Vet Sci. 2023 Aug 3;10:1223450. doi: 10.3389/fvets.2023.1223450 (PMC10435659; doi:10.3389/fvets.2023.1223450)
Supplement: Supplementary file 1 [file Presentation_1.PPTX]

## Slide 1
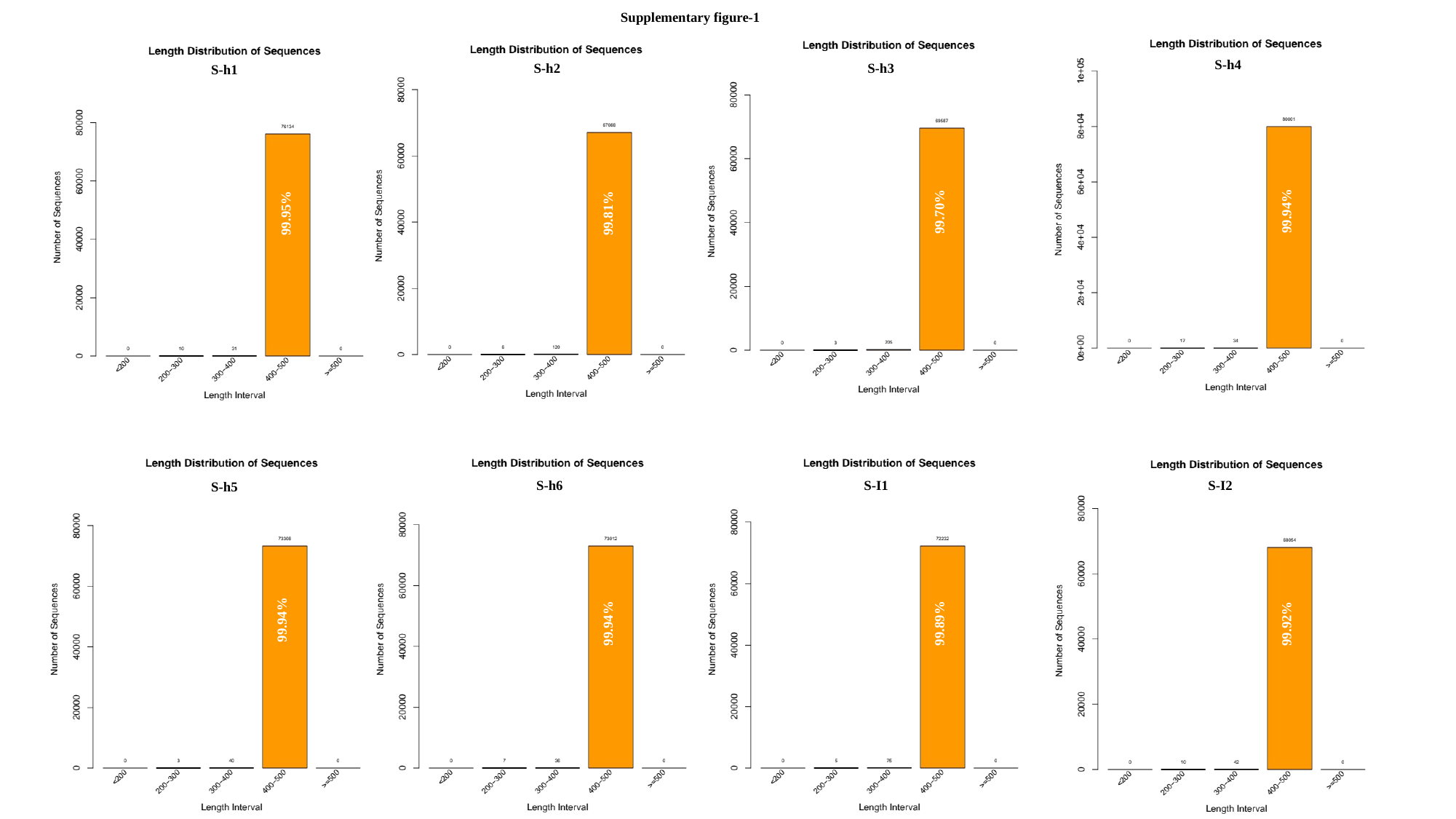

Supplementary figure-1
S-h4
S-h2
S-h3
S-h1
S-h6
S-h5
S-I1
S-I2
99.94%
99.81%
99.70%
99.95%
99.94%
99.94%
99.89%
99.92%

## Slide 2
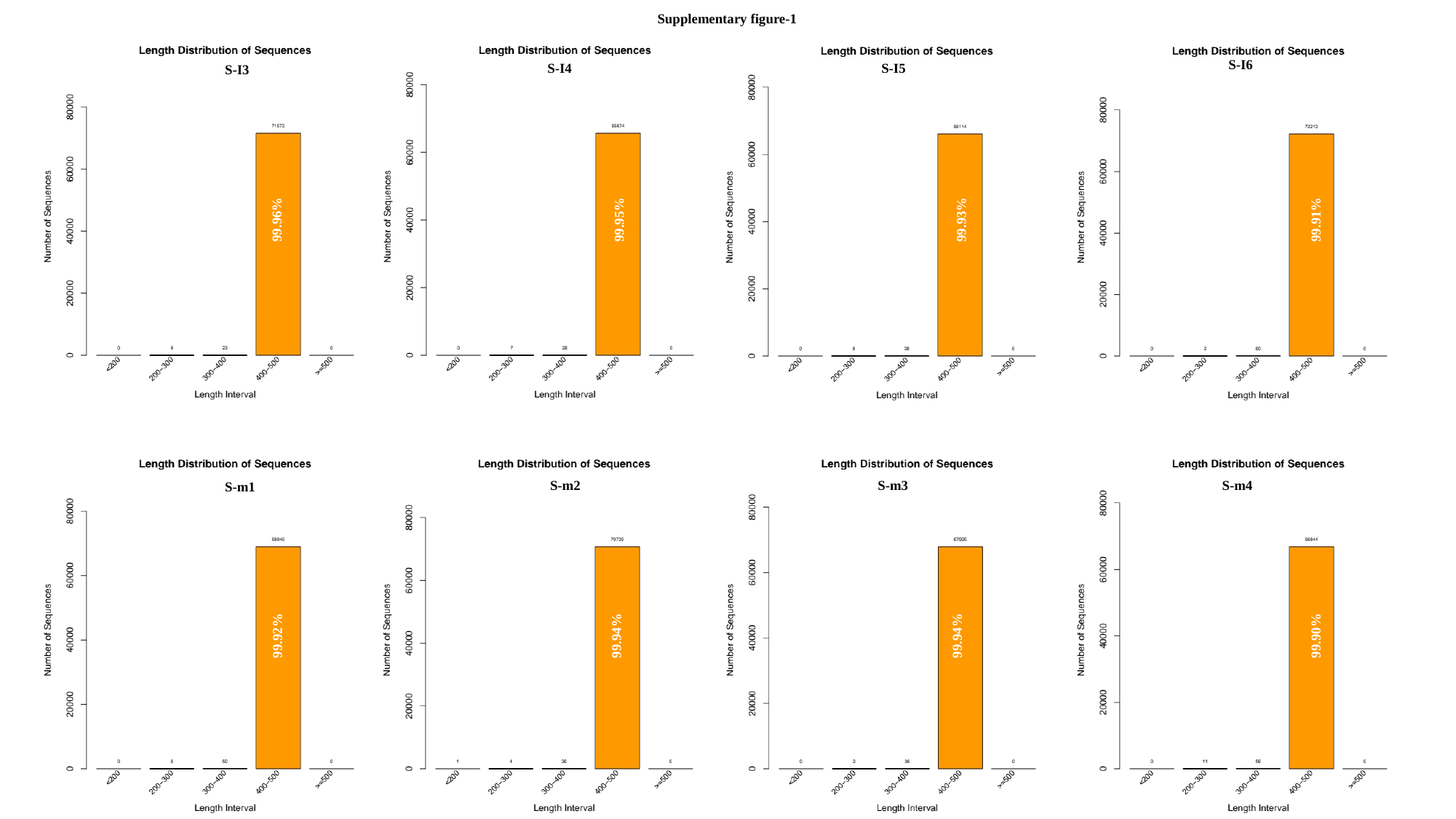

Supplementary figure-1
S-I6
S-I4
S-I5
S-I3
S-m2
S-m1
S-m3
S-m4
99.93%
99.91%
99.95%
99.96%
99.90%
99.92%
99.94%
99.94%

## Slide 3
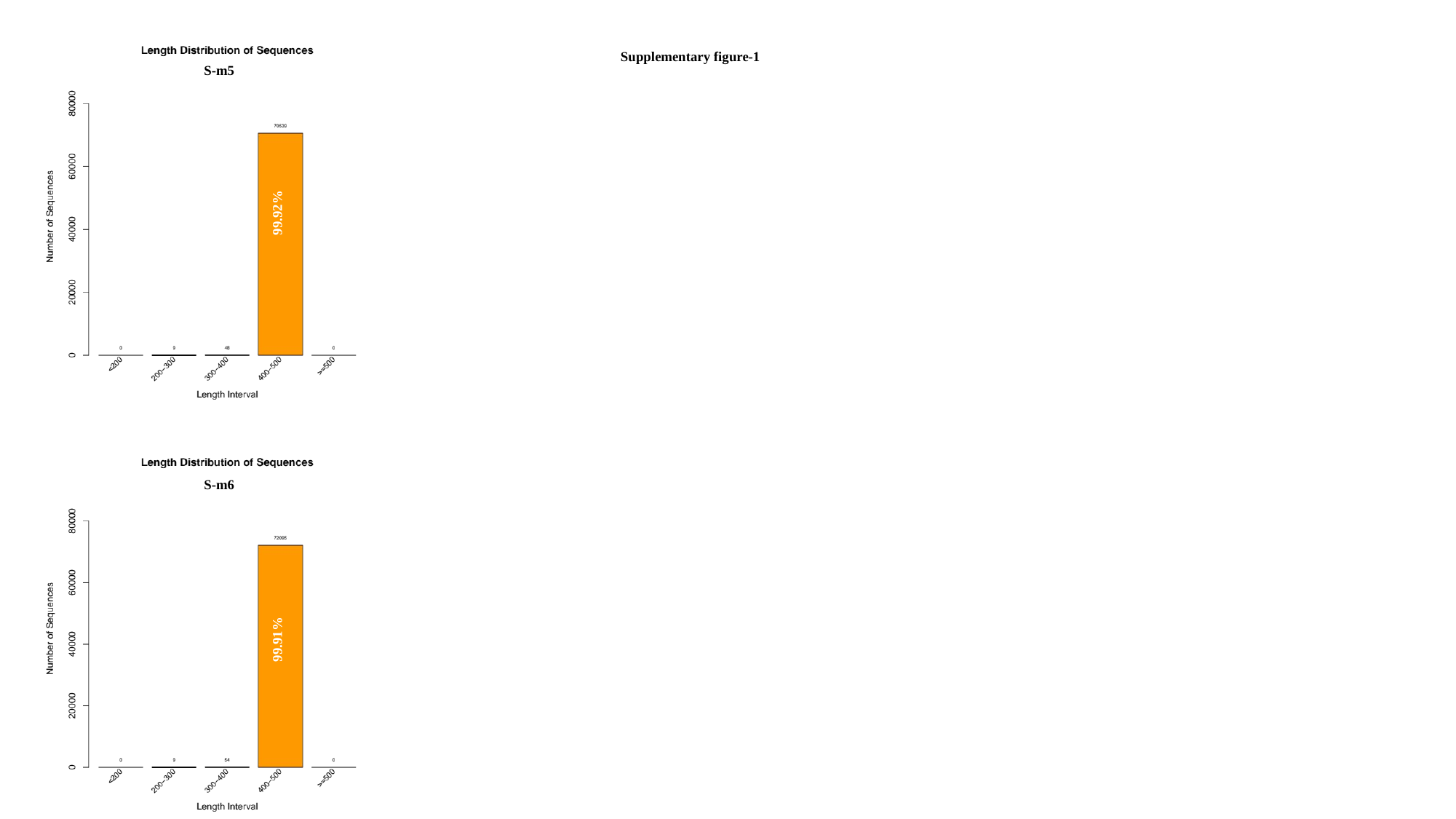

Supplementary figure-1
S-m5
S-m6
99.92%
99.9103827934964%
99.91%
